# Polymorphic variants of SLCO1B1 in neonatal hyperbilirubinemia in China

**DOI:** 10.1186/1824-7288-39-49

**Published:** 2013-08-12

**Authors:** Jiebo Liu, Jun Long, Shaofang Zhang, Xiaoyan Fang, Yuyuan Luo

**Affiliations:** 1 Department of Pediatrics, The Fifth People’s Hospital of Shenzhen, No. 47 Friendship Road, Luohu District, Shenzhen, 518001, China

**Keywords:** Polymorphisms, Neonatal hyperbilirubinemia, Case-control study, Organic anion transport polypeptide C

## Abstract

**Background:**

To evaluate the association between the genetic polymorphism of the solute carrier organic anion transporter family member 1B1 (SLCO1B1, also known as organic anion transport polypeptide C) and hyperbilirubinemia in Chinese neonates.

**Methods:**

183 infants with hyperbilirubinemia and 192 control subjects from the Fifth People’s Hospital of Shenzhen were recruited. Polymerase chain reaction, restriction fragment length polymorphisms and agarose gel electrophoresis techniques were used to detect genetic variants of SLCO1B1.

**Results:**

The study revealed that SLCO1B1 388 G > A occurred significantly more frequently in neonates with hyperbilirubinemia than in controls (RR = 1.50; 95% CI: 1.13–2.00). There were no significant differences in SLCO1B1 521 T > C between the hyperbilirubinemia and the control group (RR, 1.00; 95% CI, 0.72–1.40). No carriage of the C to A substitution at nucleotide 463 was detected.

**Conclusion:**

The SLCO1B1 388 G > A variant is associated with neonatal hyperbilirubinemia in Chinese neonates.

## Background

Neonatal hyperbilirubinemia is one of the most common problems encountered in newborns, occurring in up to 60% of healthy full-term and 80% of preterm newborns during the first week of life [1]. Most cases are physiological and do not result in serious consequences, but systematic failure to identify at-risk infants has been associated with an increased risk of severe hyperbilirubinemia, which is one of the reasons for the higher incidence of kernicterus, and may result in neurodevelopmental abnormalities such as hearing loss, athetosis, and rarely, intellectual deficits [2].

Bilirubin, the hydrophobic end product of heme degradation, is metabolized in the hepatocyte to hydrophilic conjugates, which are then efficiently eliminated in the bile [3]. Following uptake from the plasma, unconjugated bilirubin is transported from the blood circulation to the liver by organic anion transporter polypeptide 2 (OATP2), also known as OATP-C and LST1, which is an uptake transporter located on the basolateral (sinusoidal) membrane of human hepatocytes, encoded by the gene of the solute carrier organic anion transporter family member 1B1 (SLCO1B1) [4]. At this point, OATP1B1 is conjugated with glucuronic acid and bilirubin in the endoplasmic reticulum through a catalytic reaction involving UDP-glucuronosyltransferase 1A1 (UGT1A1), which results in conjugated bilirubin, also known as water-soluble bilirubin. The conjugated bilirubin glucuronides are excreted in the bile, pass through the intestine, and are deconjugated by bacteria that reduce some of the bilirubin to urobilinogens. Approximately 80% of the urobilinogens are excreted in the stool and the remainder is either reabsorbed into the enterohepatic circulation or goes to the kidneys to be excreted in urine [3]. Thus, OATP1B1 or UGT1A1 are responsible for bilirubin conjugation, and it follows that a defect in the function of either may result in unconjugated hyperbilirubinemia [5].

In our previous study, we found that the UGT1A1 211G > A mutation is associated with neonatal hyperbilirubinemia in Asians [6]. Recently, studies have suggested that variations of 388 G > A, 521 T > C, 463 C > A of the SLCO1B1 gene may predispose subjects to neonatal hyperbilirubinemia by limiting hepatic bilirubin uptake [7]. A high prevalence of 388 G > A (73.4%) and 521 T > C (14.0%) variants occurring in Chinese subjects [8]. A 16% prevalence of 463 C > A variants has been reported in Europeans and Americans, but this variant appear to be of low prevalence in the Chinese population [9]. Moreover, neonatal hyperbilirubinemia is known to occur more frequently and to be more severe in Asians than in Caucasians [10]. We hypothesized that the SLCO1B1 mutation may be one of the risk factors for neonatal hyperbilirubinemia, which possibly accounts for the variability in prevalence rates in the Chinese neonates. Therefore, we conducted a case-control study of three variants (388 G > A, 521 T > C, 463 C > A) of SLCO1B1 and investigated their association with neonatal hyperbilirubinemia in the Chinese neonates.

## Methods

### Patients and controls

The subjects were term newborn infants, with a gestational age of more than 37 weeks, who were born between August 2011 and January 2012 at the Fifth People’s Hospital of Shenzhen, China. We excluded newborn infants who were birth asphyxia, polycythemia, hypothermia, hypoglycemia, cephalohematoma, hypothyroidism, glucose-6-phosphate dehydrogenase (G-6-PD) deficiency, or hemolytic disease of ABO and Rh incompatibilities. We also excluded newborn infants whose mothers were maternal diabetes, hypertension in pregnancy, intrauterine infection.

Neonatal hyperbilirubinemia was defined as neonates with total serum bilirubin levels exceeding the 95th percentile, according to the Bhutani nomogram [11]. Total serum bilirubin levels were monitored and the causes of neonatal hyperbilirubinemia were investigated and treated following the 2004 American Academy of Pediatrics (AAP) guidelines. The control group consisted of neonates with total serum bilirubin levels below the 40th percentile as defined by the Bhutani nomogram [11]. The case-control study included a total of 183 infants with hyperbilirubinemia and 192 control subjects.

### Molecular analysis

The polymerase chain reaction–restriction fragment length polymorphism (PCR-RFLP) method was applied to detect three known variant sites (nucleotides 388, 463 and 521) of the SLCO1B1 gene. The natural or mutagenic primers, restriction enzymes, and digested restriction fragment sizes of the five known variants are listed in Table 1. The PCR mixture (20 μL) consisted of 200 ng of DNA, 20 ng of each primer, 1.25 mM of each dNTP (3.2 μL), buffer solution (100 mM Tris HCl, 500 mM KCl, 15 mM MgCl_2_, and 0.01% gelatin), and 0.4 U of Taq DNA polymerase. The PCR amplification was carried out in a thermal cycler for 35 cycles of denaturation for 1 min at 94°C, annealing for 1 min at 55°C, primer extension for 1 min at 72°C, and a final extension for 10 min at 72°C. The PCR product was digested with the appropriate restriction enzyme and analyzed on a 3% agarose gel.

**Table 1:** Natural or mutagenesis primers, restriction enzymes, and SLCO1B1 genes and variations

**Positions (cDNA)**	**Primers**	**Sequences**	**Restriction enzymes**	**Results (bp)**
388	388 F	5′ATAATGGTGCAAATAAAGGGG3′	Taq I	G 128 + 63 + 23
G → A	388R	5′ACTATCTCAGGTGATGCTCTA3′		A 151 + 63
521	521 F	5′TTGTCAAAGTTTGCAAAGTG3′	Hha I	T 209
T → C	521R	5′GAAGCATATTACCCATGAGC3′		C 189 + 20
463	463 F	5′ATAATGGTGCAAATAAAGGGG3′	Hpa II	C 205 + 19
C → A	463R	5′ACCTTTTCCCACTATCTCCG3′		A 224

### Statistical analysis

We analyzed the collected data using Stata software (version 9.0; Stata Corp. LP, College Station, TX, USA). Genotype results were analyzed as the hyperbilirubinemic group versus the control group. Allele frequencies of genes were determined by calculating the percentage of variant genes within the total number in the study population. Comparisons between the study group and the control group were performed with chi-square test and Fisher’s exact test when expected values were <5%. Results are given as relative risk (RR) with 95% confidence intervals (CI).

### Ethical considerations

The Ethics Committee on Human Experimentation of the Faculty of Medicine, Fifth People’s Hospital of Shenzhen, China, approved the study protocol. Informed consent was obtained from the parents of each study infant.

## Results

A total of 375 neonates were enrolled into the study. Newborns with G6PD deficiency were not found in the study group. The demographic and clinical data of the hyperbilirubinemic group and the control group is summarized in Table 2. There were no statistically significant differences in gestational age, birth weight, sex, delivery, feeding between the two groups.

**Table 2:** Demographic and clinical data of newborn infants enrolled into the study

**Data**	**Hyperbilirubinemia group (n = 183)**	**Control group (n = 192)**	**p-value**
Gestational age (week)	38.35 ± 1.76	38.12 ± 1.53	0.18
Birth weight (g)	3238.32 ± 409.37	3194.53 ± 399.61	0.29
Sex: male	98 (53.55%)	102 (53.12%)	0.98
Delivery:			
Normal	118 (64.48%)	121 (63.02)	0.85
Cesarean section	45 (24.59%)	49 (25.52%)	0.93
Forceps and vacuum extraction	20 (10.93%)	22 (11.46%)	0.99
Feeding:			
Breast milk only	46 (25.14%)	54 (28.13%)	0.59
Formula mixed with breast milk	94 (51.37%)	97 (50.52%)	0.95
Formula only	43 (23.49%)	41 (21.35%)	0.71
Excessive weight loss (>3% per day or ≥10% of birth weight)	112 (61.2%)	119 (61.98%)	0.96

Analysis of SLCO1B1 at nt 388 revealed that 76 of the 183 (41.53%) neonates in the hyperbilirubinemic group demonstrated the transition mutation (17 homozygous and 59 heterozygous) compared to 53 of 192 (27.60%) in the control group (10 homozygous and 43 heterozygous). There were significant differences between genotype distributions of the SLCO1B1 388 G > A mutation (Table 3). The risk of neonatal hyperbilirubinemia was higher in SLCO1B1 388 G > A allele carriers (A/A + G⁄A) than in G⁄G allele carriers (RR, 1.50; 95% CI, 1.13–2.00).

**Table 3:** Distributions of SLCO1B1 genotypes and allele frequencies with variants at nucleotide 388, 521 and 463

**Variants of genes**	**Group**	**Homozygous wild type**	**Heterozygous**	**Homozygous mutant type**	**Allele frequency**	**p-value**
SLCO1B1	Case	107	59	17	0.254	
nt 388	Control	139	43	10	0.164	0.003
SLCO1B1	Case	137	35	11	0.156	
nt 521	Control	141	42	9	0.155	0.937
SLCO1B1	Case	183	0	0	0	
nt 463	Control	192	0	0	0	-

The allele frequency of the variant SLCO1B1 at nt 388 in infants in the hyperbilirubinemic group was 0.254, which was significantly higher than that observed in the control group (0.164, p = 0.003; Table 3). The likelihood of developing neonatal hyperbilirubinemia was 1.55 times higher in infants with the A allele in SLCO1B1 388 G > A than in infants with the G allele (95% CI, 1.16–2.06).

Analysis of the variant of SLCO1B1 gene at nt 521 revealed an allele frequency of 46 of 183 (25.14%) in neonates in the hyperbilirubinemic group (11 homozygous and 35 heterozygous), compared to 51 of 192 (26.56%) in the control group (9 homozygous and 42 heterozygous). There were no statistically significant differences in the risk of neonatal hyperbilirubinemia between SLCO1B1 521 T > C allele carriers (C/C + C/T) and T⁄T allele carriers (RR, 0.95; 95% CI, 0.67–1.34).

The allele frequency of the variant SLCO1B1 at nt 521 in infants in the hyperbilirubinemic group was 0.156, compared with 0.155 in the control group (Table 3). There were no statistically significant differences in the risk of neonatal hyperbilirubinemia between those with the T allele in SLCO1B1 521 T > C and those with the C allele (RR, 1.00; 95% CI, 0.72–1.40). No carriage of the C to A substitution at nucleotide 463 was detected (Table 3).

## Discussion

Various factors are involved in the development of neonatal hyperbilirubinemia, including the SLCO1B1 gene, which requires further investigation as SLCO1B1 encodes a liver-specific member of the organic anion transporter family. The encoded protein is a transmembrane receptor that can rapidly and selectively uptake bilirubin into hepatocytes [12]. Variations within the coding region of the SLCO1B1 gene may result in dysfunction of SLCO1B1 due to abnormal structure [13]. In adult liver disease, polymorphism in the gene encoding this protein is a major determinant of serum bilirubin levels [14]. These findings indicate that variations in the SLCO1B1 gene are possibly associated with neonatal hyperbilirubinemia. The most frequent mutations of SLCO1B1 in the Chinese population are the 388 G > A mutation and the 521 T > C mutation [15]. The prevalence of 463 C > A variants of SLCO1B1 gene is low in Chinese, but high in Europeans and Americans [16]. Neonatal hyperbilirubinemia is more frequent and more severe in Chinese than in Europeans and Americans [17], and the SLCO1B1 mutation may explain the variability in the prevalence of neonatal hyperbilirubinemia among different ethnic groups.

Our study revealed that the 388 G > A mutation of the SLCO1B1 gene is associated with neonatal hyperbilirubinemia in Chinese neonates. Assessment of the frequency of the G allele in the 388 G > A mutation in hyperbilirubinemic and in control infants (0.254 and 0.164, respectively) revealed significant differences. The likelihood of developing neonatal hyperbilirubinemia was 1.55 times higher in 388 G > A mutants with the A allele than in those with the G allele. The allele frequency of the variant SLCO1B1 at nt 521 in infants in the hyperbilirubinemic group did not demonstrate a statistically significant difference compared with the control group (0.156 and 0.155, respectively). No carriage of the C to A substitution at nucleotide 463 of the SLCO1B1 gene was detected. The results of our case-control study were consistent with those of a previous study involving the Chinese neonates, which is conflicting with the study from Brazil [18]. The allele frequency of SLCO1B1 (388A > G) in chinese populations was 63.5% [19], which was higher than Brazilians (white was 50.4% and black was 35.1%) [20]. The genetics of racial differences might explain the different association between the genetic polymorphism of SLCO1B1 and neonatal hyperbilirubinemia among different ethnic groups.

In genetic epidemiology, a genome-wide association study (GWAS) examines all or most of the genes of different variations associated with different diseases. It is more powerful than a case-control study for single genetic polymorphisms. Recently, one GWAS based on European adult populations suggested that genetic variations of SLCO1B1 influence bilirubin levels [21]. Another cohort from the Mayo Genome Consortia also showed that genetic variants of SLCO1B1 were associated with total bilirubin levels in Americans [22]. However, both a GWAS based on European adult populations [23] and a large-scale GWAS based on Korean adults [24] contradicted these results, suggesting that genetic variations of SLCO1B3 influence bilirubin levels, not SLCO1B1. The SLCO1B3 gene is also a member of the OATP family, which is highly expressed in the basolateral membrane of hepatocytes. SLCO1B3 and SLCO1B1 share 80% amino acid sequence identity and have similar substrate selectivity [25]. Functionality between these two human transporters in the OATP family can be distinguished using estrone-3-sulfate, a SLCO1B1-selective ligand, and cholecystokinin octapeptide, a SLCO1B3-selective ligand [26]. Whether SLCO1B3 and SLCO1B1 are associated with bilirubin levels or differentially contribute in a population-specific fashion, needs further investigation.

## Conclusions

The cohort study which analyzed the genetic association was time-consuming, laborious, and high costly, so, there was still not one prospective cohort study which assessed genetic variations associated with hyperbilirubinemia. Our case-control study found that the variant SLCO1B1 388 G > A is associated with neonatal hyperbilirubinemia in Chinese neonates; further prospective cohort study is need to conform the association.

## Competing interests

The authors declare that they have no competing interests.

## Authors’ contributions

JL, SZ carried out the molecular genetic studies; JL, XF participated in the sequence alignment; JL, YL design of experiment and drafted the manuscript; SZ, XF collected and interpreted the data; JL, SZ wrote the manuscript; JL, SZ, XF, YL reviewed the Manuscript. All authors read and approved the final manuscript.
